# Seasonal impacts on *Salmonella* prevalence and antimicrobial resistance in the pork food chain of northeastern Thailand

**DOI:** 10.1128/aem.00057-26

**Published:** 2026-06-02

**Authors:** Hongmei Liu, Zhongbin Bai, Ning Wang, Chaiwat Pulsrikarn, Patchara Phuektes, Prawit Butudom, Yaowen Liu, Weidong Zuo, Renchun Ji, Tingkai Bi, Chunfen Mei, Rong Zhou, Wengui Li, Yi Wu, Xiaowei Sun, Yiduan Liu, Jamal Muhammad Khan, Sunpetch Angkititrakul, Fanan Suksawat, Xin Wu

**Affiliations:** 1 Faculty of Veterinary Medicine, Khon Kaen University 26684, Khon Kaen, Thailand; 2 Yunnan Joint International R&D Center of Veterinary Public Health, College of Veterinary Medicine, Yunnan Agricultural University 12616, Kunming, China; 3 Department of Medical Sciences, Ministry of Public Health, National Institute of Health, Nonthaburi, Thailand; 4 Kunming Customs Technology Center, Kunming, Yunnan, China; 5 Yunnan Animal Husbandry Station, Kunming, China; 6 Department of Parasitology, Cholistan University of Veterinary and Animal Sciences 638978, Bahawalpur, Pakistan; Anses, Maisons-Alfort Laboratory for Food Safety, Maisons-Alfort, France

**Keywords:** seasonal impacts, *Salmonella*, prevalence, antimicrobial resistance, pork chain

## Abstract

*Salmonella* remains a critical foodborne pathogen of public health importance and a predominant contributor to contamination throughout the pork production system. However, in northeastern Thailand, detailed characterization of seasonal variation and stage-specific occurrence, including pre-slaughter fecal samples, post-slaughter carcasses, and retail pork, has been largely lacking. Clarifying the influence of seasonal conditions on *Salmonella* prevalence and antimicrobial resistance across these production points is essential for robust risk assessment within the pork supply chain. A total of 897 samples were collected from slaughterhouses (carcasses, feces) and markets (pork) in northeastern Thailand during the summer, rainy, and winter seasons from 2023 to 2024. *Salmonella* isolation, serotyping, antimicrobial susceptibility testing, pulsed-field gel electrophoresis (PFGE), and phylogenetic analysis were performed to assess prevalence, serotype distribution, AMR, and genetic relatedness. The associations between prevalence or AMR and seasonal factors were evaluated using relative risk (RR), chi-square, and logistic regression tests. *Salmonella* prevalence varied markedly across matrices and seasons. Retail pork showed the highest contamination (summer: 75.4%, rainy: 75.3%, winter: 54.7%), followed by feces (50.5%, 34.0%, and 29.0%, respectively), whereas carcass samples had the lowest prevalence (11.3%, 13.0%, and 4.0%). Seasonal effects were significant for fecal (χ² = 11.4, *P* = 0.003) and pork samples (χ² = 7.7, *P* = 0.021). Relative risk (RR), using winter as reference, reached 1.7 for fecal samples and 1.4 for pork during summer. Serotype composition exhibited pronounced seasonal shifts, with *S*. Rissen increasing sharply and becoming dominant in winter; logistic regression showed significantly lower odds of *S*. Rissen isolation in summer (OR = 0.25, *P* < 0.01) and the rainy season (OR = 0.47, *P* < 0.01) compared with winter. Multivariable models indicated that the rainy season was strongly associated with antimicrobial resistance (OR = 11.9 vs winter, *P* = 0.002). *S*. Rissen remained the primary contributor to MDR across matrices, while *S*. Bovismorbificans and other *serotype*s showed significantly reduced resistance (OR = 0.2, *P* < 0.05). PFGE analyses revealed high polymorphism in *S*. Weltevreden (55%–100% similarity) and *S*. Rissen (50%–100%), with no clustering by matrix or season, indicating cross-environmental dissemination of similar clones. Phylogenetic analysis identified two major *S*. Rissen lineages but a highly conserved MDR gene backbone, including β-lactamases (blaTEM-1, blaCTX-M-55), aminoglycoside resistance genes (aadA, aac(6′)-Iy), phenicol resistance genes (cmlA6, floR), sulfonamide and trimethoprim resistance genes, and PMQR determinants (qnrB10, qnrS1). *Salmonella* contamination and antimicrobial resistance in the pork production chain are strongly shaped by seasonal pressures and downstream amplification during distribution. Summer and the rainy season present the highest risk for both contamination and multidrug resistance, while winter shows marked serotype convergence, dominated by *S*. Rissen. Retail pork exhibited the most complex MDR profiles, indicating an accumulation of resistance along the supply chain. PFGE and phylogenetic analyses revealed that closely related clones circulate across slaughterhouses and markets, supporting persistent transmission routes. These findings highlight the need for strengthened seasonal risk management and integrated genomic surveillance to interrupt the spread of high-risk MDR serotypes and reduce consumer exposure.

**IMPORTANCE:**

Understanding when and how *Salmonella* spreads along the pork production chain is essential for reducing human exposure to foodborne pathogens and multidrug-resistant (MDR) strains. This study identifies strong seasonal pressures and downstream amplification during distribution as key drivers of *Salmonella* contamination and antimicrobial resistance. The marked winter dominance of MDR *S*. Rissen and the accumulation of complex resistance profiles in retail pork highlight predictable high-risk periods and critical control points where interventions can be most effective. Furthermore, the discovery that closely related clones circulate between slaughterhouses and markets underscores persistent transmission routes that conventional hygiene measures may fail to interrupt. By integrating epidemiological, phenotypic, and genomic evidence, this work provides actionable insights for designing season-specific monitoring programs and implementing genomic surveillance to limit the spread of MDR *Salmonella* in the food supply.

## INTRODUCTION

Foodborne illnesses remain a significant global public health concern (1), with *Salmonella* species among the leading causative agents. According to the World Health Organization’s most recent estimates, approximately 600 million people fall ill from unsafe food each year, and about 420,000 die as a result (2). Historically, the non-typhoidal *Salmonella* serotypes *S*. Typhimurium and *S*. Enteritidis dominated global infection burdens, particularly from the late 20th century through the early 21st century (3). However, recent surveillance has further highlighted that other serotypes are undergoing changing prevalence patterns; notably, *Salmonella enterica* serotype Rissen (*S*. Rissen) has shown a rising trend in prevalence within the global pork production chain (4). According to nationwide surveillance studies in China and similar findings from food-chain monitoring in Europe, the relative proportion of serotypes such as *S*. Rissen has been steadily increasing (5).

In Thailand, the prevalence and incidence of *Salmonella* infections have remained markedly high according to national surveillance and epidemiological studies (6). Pork—a dietary staple—has consistently been implicated as a major reservoir. Studies have reported contamination rates as high as 80% in retail meats and over 40% in pork samples from slaughterhouses and markets. Remarkably, *S*. Rissen has recently surpassed traditionally dominant serotypes like *S*. Typhimurium, *S*. Anatum, and *S*. Stanley, becoming the most frequently isolated serotype in northern Thailand (49% of pig fecal isolates) and across southern provinces, slaughterhouses, and retail markets (7, 8).

The increasing dominance of *S*. Rissen raises critical questions about the underlying drivers of this epidemiological shift. Whether this transition is linked to ecological and environmental factors—such as seasonal fluctuations in temperature and humidity—or to changes in farming and food distribution practices remains unclear (9). Compounding this concern, *S*. Rissen isolates in Thailand frequently exhibit multidrug resistance (MDR), particularly against first-line antibiotics such as ampicillin, tetracycline, and streptomycin, with resistance rates exceeding 80–90% in some regions (10, 11). Given that antimicrobials remain a cornerstone in the treatment of invasive salmonellosis, the rise of MDR *S*. Rissen poses a substantial challenge to clinical management and food safety (12). Despite its recognized importance, comprehensive studies that integrate prevalence data, antimicrobial resistance profiles, and molecular epidemiology of *S*. Rissen across different seasons and sources are still lacking.

To address these gaps, this study investigates how seasonal variation shapes the prevalence, serotype distribution, and antimicrobial resistance patterns of *Salmonella* along the slaughterhouse-to-retail pork production chain in northeastern Thailand, with a particular focus on *S*. Rissen. By integrating phenotypic resistance profiles with whole-genome-based phylogenetic analyses, this research aims to elucidate the ecological and epidemiological drivers facilitating the dissemination of MDR *S*. Rissen. The findings are expected to provide evidence for targeted interventions that enhance slaughterhouse hygiene, strengthen seasonal risk management, and support genomic surveillance strategies to protect public health.

## MATERIALS AND METHODS

### Sample size calculation

The sample size for this study was determined using simple random sampling methods and formulas based on different seasons:


$$n=\frac{{(1.96)}^{2}P(1-P)}{L^{2}}$$

The expected prevalence of the sample is approximately 25.00% (13). The 95% confidence interval (CI) and allowable error were set to be 5.00%. The sample size for each season was 288. Each sample type represented approximately one-third of the total number of samples.

### Sample collection

A total of 897 samples were collected from slaughterhouses and markets in northeastern Thailand between April 2023 and February 2024, covering three climatic seasons. Sampling was conducted in Udon Thani and Khon Kaen, selected based on their representation of the pork production system in the region, as follows.

- Geographic and economic relevance: Khon Kaen is a central hub for commercial pig farming and slaughtering, while Udon Thani serves as a key distribution point for pork products toward the Laos border.
- Complete pork supply chain: Both provinces include farms, slaughterhouses, and markets, providing a comprehensive view of *Salmonella* prevalence and antimicrobial resistance across the production and retail environments.
- Seasonal variation: Samples were collected across three climatic seasons to assess seasonal impacts on *Salmonella* and antimicrobial resistance.

These criteria ensure that the samples are representative of the pork production system in northeastern Thailand, allowing for a thorough evaluation of contamination and resistance patterns.

Sampling was conducted at one standardized slaughterhouse and three central traditional markets in Udon Thani and Khon Kaen. These sites were selected based on slaughter volume, supply-chain coverage, and representativeness of typical commercial and retail settings, as follows.

- Slaughterhouse selection: Two standardized slaughterhouses were chosen based on a minimum daily slaughter volume threshold to represent large-scale commercial operations.
- Market selection: Three central markets in each province were selected based on market volume and customer traffic, serving as downstream retail points of the slaughterhouse supply chain, thus representing typical consumer-facing settings.
- Sampling frequency: Sampling was conducted twice per season at both slaughterhouses and retail markets to ensure seasonal coverage.

In summer (March–May; humidity 58–77%, temperature 22–34°C; *n* = 283), 115 carcass swabs and 111 fecal samples were collected from slaughterhouses, while 57 pork samples were obtained from retail markets. In the rainy season (June–October; humidity 83–86%, temperature 24–30°C; *n* = 297), 100 carcass swabs and 100 fecal samples were collected, together with 97 pork samples from retail markets. In winter (November–February; humidity 59–68%, temperature 17–30°C; *n* = 317), 100 carcass swabs and 100 fecal samples were obtained from slaughterhouses, and 117 pork samples were collected from retail markets.

### Isolation and identification of *Salmonella* spp.

*Salmonella* isolation and identification were performed following ISO 6579:2002/AMD 1:2007. Each carcass swab, fecal swab, and pork swab was transferred into 9 mL of buffered peptone water (BPW) for pre-enrichment and incubated at 37°C for 18–24 h.

Following pre-enrichment, a loopful of the BPW culture was streaked onto modified semi-solid Rappaport-Vassiliadis (MSRV) agar and incubated at 41.5–42°C for 18–24 h. From the migration zone on MSRV, three typical colonies were selected and streaked onto xylose lysine deoxycholate (XLD) agar. After incubation at 37°C for 24 h, presumptive *Salmonella* colonies (red colonies with black centers) were chosen for biochemical confirmation. Biochemical identification was performed using triple sugar iron (TSI) agar and motility-indole-lysine (MIL) agar. Colonies that exhibited alkaline slant/acid butt with H₂S production in TSI and motility with lysine decarboxylation in MIL were considered presumptive *Salmonella* spp. Only isolates fulfilling both biochemical criteria were retained for further characterization.

### Antimicrobial resistance testing

Antimicrobial susceptibility was assessed using the Kirby–Bauer disk diffusion method following CLSI guidelines. The tested antibiotics included AMC (20 μg), AMP (10 μg), CTX (30 μg), CAZ (30 μg), CRO (30 μg), SXT (1.25/23.75 μg), IMP (10 μg), GEN (10 μg), STP (10 μg), NAL (30 μg), NOR (30 μg), CIP (5 μg), TET (30 μg), and CHL (30 μg). Bacterial suspensions were adjusted to 0.5 McFarland standard and spread on Mueller–Hinton agar. Disks were applied within 15 min, and plates were incubated at 37°C for 18–24 h. Inhibition zones were measured and interpreted based on CLSI breakpoints. *E*. coli ATCC 25922 served as the quality control.

### Serotype

Serotype identification was conducted at the Department of Medical Science, Ministry of Public Health, Bangkok, Thailand. The slide agglutination method was carried out with the commercial antisera according to the manufacturer’s instructions (S&A Reagents Ltd., Bangkok, Thailand) to detect the presence of O (somatic) and H (flagellar) antigens. Based on the positive results of O and H antigens, the results were interpreted following the White-Kauffmann-Le Minor scheme (14).

### Pulsed-field gel electrophoresis (PFGE)

The PFGE follows the international PulsNet *Salmonella* PFGE standard experimental protocol (15). Strain selection was based on 20 strains of each dominant serotype under each season. Agarose plugs containing genomic DNA from each isolate were prepared and subjected to overnight digestion with 24 U of *Xba*I enzyme (Promega). The resulting restriction fragments were separated on a 1% agarose gel (Bio-Rad Certified Agarose) in 0.5 × TBE buffer using a CHEF III system (Bio-Rad). To ensure DNA integrity, 50 μM thiourea was added to the running buffer. Electrophoresis conditions included a voltage gradient of 6 V/cm, a 120° angle, an initial switch time of 2 s, a final switch time of 64 s, and a total run time of 22 h. *Salmonella* Braenderup H9812 *Xba*I-digested DNA served as a standardized molecular reference marker. The obtained PFGE profiles were analyzed using GelCompar II 5.0 software (Applied Maths), applying the Dice coefficient for comparison and clustering with the unweighted pair-group method with arithmetic averages (UPGMA) using 1% optimization and 0.5% band position tolerance.

### Whole-genome sequencing

Whole-genome sequencing (WGS) of selected *Salmonella* Rissen isolates was conducted by Sangon Biotech (Shanghai, China). Nine isolates of the dominant serovar in this study (*S*. Rissen) were selected for WGS, with three isolates randomly chosen from each season. This per-season random selection was intended to minimize isolate-to-isolate variation and to better capture season-associated genomic signals.

Genomic DNA was extracted using a magnetic bead-based method. DNA quality and concentration were assessed using the Qubit Fluorometer Nucleic Acid Quantification Instrument and agarose gel electrophoresis. Sequencing libraries were prepared following Illumina protocols and sequenced on the Illumina NovaSeq 6000 platform, generating 2 × 150 bp paired-end reads. Raw sequencing data underwent quality control using FastQC v0.11.2, and low-quality reads and adapter sequences were trimmed using Trimmomatic v0.36. High-quality reads were *de novo* assembled using SPAdes v3.5.0, and genome annotation was performed using Prokka v1.10. Pan-genome analysis was conducted using Roary v3.13.0 to identify core and accessory genes among isolates. Single-nucleotide polymorphisms (SNPs) were identified using Snippy v4.6.0, and a maximum-likelihood phylogenetic tree was constructed with RAxML v8.2.12 under the GTRGAMMA model with 1,000 bootstrap replicates. Antimicrobial resistance (AMR) genes were detected using ResFinder 4.1 with default parameters (identity ≥90%, minimum length ≥60%), and plasmid replicons were screened using PlasmidFinder.

### Statistical analysis

All statistical analyses were performed using SPSS version 18.0 (SPSS Inc., Chicago, IL, USA). Prevalence differences of *Salmonella* among seasons and sample types were evaluated using chi-square tests, and relative risk (RR) with 95% confidence intervals were calculated for pairwise seasonal comparisons. Serotype distribution across seasons was examined using chi-square tests, followed by univariable logistic regression to estimate odds ratios (ORs) for dominant serotypes relative to the reference category. For antimicrobial resistance (AMR), multivariable logistic regression models were applied to assess the independent effects of season, sample matrix, and serotype on the likelihood of resistance, using winter, carcass samples, and *S*. Rissen as reference categories. A two-tailed *P*-value < 0.05 was considered statistically significant.

## RESULTS

### Seasonal and matrix-specific prevalence of *Salmonella* spp.

**Fig 1:**
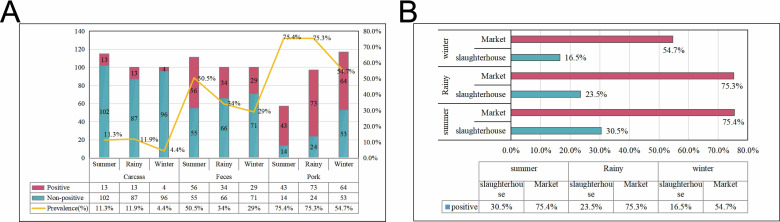
Seasonal and matrix/site-specific prevalence of *Salmonella* in samples collected from slaughterhouses and markets in northeastern Thailand. (**A**) Prevalence of *Salmonella* in different sample matrices (carcass, feces, and pork) across three seasons. (**B**) Prevalence of *Salmonella* in slaughterhouse and market samples during different seasons.

Seasonal variation in *Salmonella* prevalence was assessed across three sample matrices representing distinct stages in the pork production chain: carcass swabs and fecal samples collected at slaughterhouses, and pork swabs collected from retail markets (Fig. 1A). Prevalence varied across matrices and seasons. In carcass samples, the highest prevalence was observed in the rainy season at 13.0%, followed by summer at 11.3% and winter at 4.0%. Fecal samples showed higher contamination, with rates of 50.5% in summer, 34.0% in the rainy season, and 29.0% in winter. Pork samples collected from retail markets exhibited the highest prevalence, with 75.4% in summer, 75.3% in the rainy season, and 54.7% in winter. In summary, carcass samples had the lowest prevalence, while pork samples from retail markets had the highest.

When stratified by sampling site (Fig. 1B), retail pork consistently showed substantially higher contamination levels than slaughterhouse samples in all seasons. In summer, *Salmonella* was detected in 30.5% of slaughterhouse samples versus 75.4% of market samples; in the rainy season, the corresponding values were 23.5% and 75.3%, and in winter 18.0% and 54.7%, respectively, indicating an accumulation of contamination downstream in the distribution chain.

Seasonal effects were further evaluated within each matrix using chi-square analysis (Table 1). Carcass samples showed no significant seasonal variation (χ² = 3.9, *P* = 0.14). In contrast, fecal samples demonstrated a significant seasonal effect (χ² = 11.4, *P* = 0.003), with higher prevalence in summer compared with the rainy (*P* = 0.016) and winter seasons (*P* = 0.002). Pork samples also showed significant seasonal variation (χ² = 7.7, *P* = 0.021); pairwise comparisons indicated that both summer and rainy seasons had significantly higher prevalence than winter (*P* = 0.025 and *P* = 0.009, respectively), while no difference was observed between summer and the rainy season (*P* = 0.994).

**TABLE 1:** Seasonal variation in *Salmonella* prevalence across carcass, fecal, and pork samples with chi-square test results^*a*^

Sample category	Season	Positive	Non-positive	Prevalence (%)	*χ* ^2^	df	*P*-value
Carcass	Summer	13	102	11.3	3.9	2	0.14
	Rainy	13	87	13			
	Winter	4	96	4			
Feces	Summer	56	55	50.5	11.4	2	0.003
	Rainy	34	66	34			
	Winter	29	71	29			
Pork	Summer	43	14	75.4	7.7	2	0.021
	Rainy	73	24	75.3			
	Winter	64	53	54.7			

^*a*^ See the supplemental material. For carcass samples, no significant difference was observed between summer and rainy seasons (χ² = 0.0, *P* = 1.000). The comparison between summer and winter showed no significant difference (χ² = 2.4, *P* = 0.121), and no significant difference was also found between rainy season and winter (χ² = 2.7, *P* = 0.103). For fecal samples, significant differences were found between summer and rainy seasons (χ² = 5.8, *P* = 0.016) and between summer and winter (χ² = 10.1, *P* = 0.002). No significant difference was observed between rainy season and winter (χ² = 0.6, *P* = 0.445). For pork samples, no significant difference was observed between summer and rainy seasons (χ² = 0.0, *P* = 0.994). A significant difference was found between summer and winter (χ² = 5.0, *P* = 0.025), and the comparison between rainy season and winter also showed a significant difference (χ² = 6.8, *P* = 0.009).

Relative risk (RR) analysis using winter as the reference season (Table 2) supported the observed patterns. RR values for carcass samples were 2.8 in summer and 3.3 in the rainy season. Fecal samples showed an RR of 1.7 in summer and 1.2 in the rainy season. Pork samples exhibited RR values of 1.4 in both summer and rainy seasons. Collectively, these findings indicate higher *Salmonella* contamination risk during summer and rainy seasons, with winter representing the period of lowest prevalence across all matrices.

**TABLE 2:** *Salmonella* prevalence and relative risk (RR) among different seasons

Sample category	Season	Positive	Non-positive	Prevalence (%)	RR	*P*-value
Carcass	Summer	13	102	11.3	2.8	0.121
	Rainy	13	87	13	3.3	0.103
	Winter	4	96	4.4	1 (ref)	–^*a*^
Feces	Summer	56	55	50.5	1.7	0.002
	Rainy	34	66	34	1.2	0.44
	Winter	29	71	29	1 (ref)	–
Pork	Summer	43	14	75.4	1.4	0.0025
	Rainy	73	24	75.3	1.4	0.009
	Winter	64	24	54.7	1 (ref)	–

^*a*^ –, not applicable.

### Serotype

**Fig 2:**
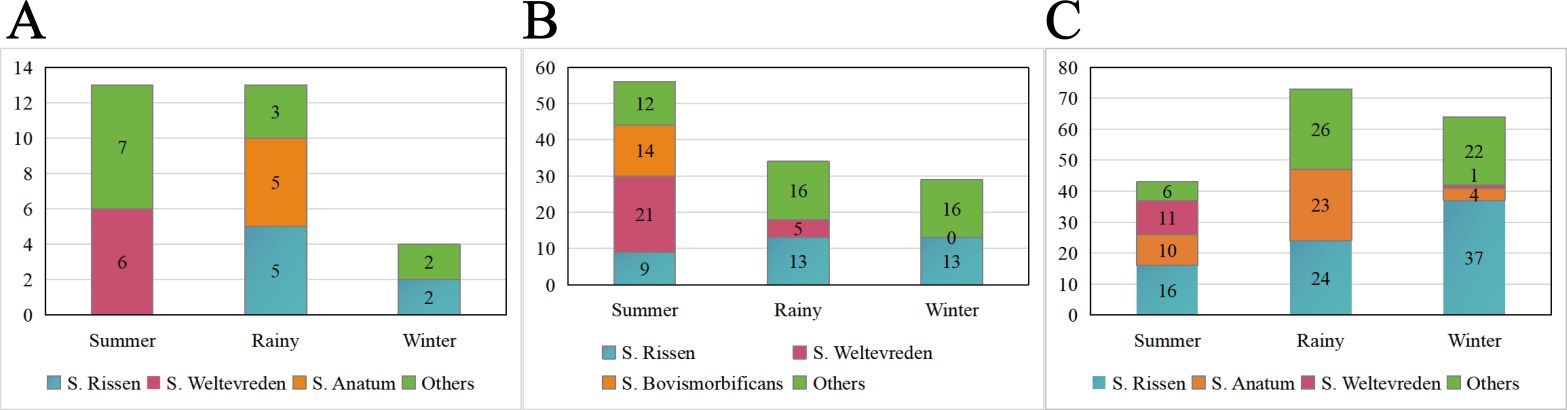
Seasonal distribution of *Salmonella* serotypes detected in carcass (**A**), fecal (**B**), and pork (**C**) samples collected from slaughterhouses and markets in northeastern Thailand. Stacked bar charts illustrate the proportional representation of major serotypes, with a specific focus on *S.* Rissen, across summer, rainy, and winter seasons.

Figure 2 illustrates a clear seasonal restructuring of *Salmonella* serotype composition, beginning with a heterogeneous distribution in summer (Fig. 2A), progressing toward partial consolidation during the rainy season (Fig. 2B), and culminating in a pronounced winter dominance of *S.* Rissen (Fig. 2C). Importantly, these visual trends are not merely descriptive; they are strongly supported by statistical analyses presented in Table 3. The proportion of *S.* Rissen varied significantly across seasons (χ² = 22.12, *P* < 0.001), mirroring the progressive increase observed in the figures. Pairwise comparisons further confirmed that the winter surge in *S.* Rissen diverged sharply from the summer (χ² = 22.09, *P* < 0.001) and rainy seasons (χ² = 7.46, *P* = 0.006). Logistic regression analyses reinforced this pattern by identifying winter as the highest-risk period (OR = 1.00, reference) and demonstrating significantly reduced odds of detecting *S.* Rissen during summer (OR = 0.25, *P* < 0.01) and the rainy season (OR = 0.47, *P* < 0.01). Together, these aligned visual and statistical signals provide compelling evidence for a structured and seasonally driven shift toward *S.* Rissen dominance.

**TABLE 3:** Seasonal variation in *S*. Rissen^*a*^

Season	*S*. Rissen	Non-*S*. Rissen	Rate	*χ* ^2^	*P*-value (*χ^2^*)	OR	*P*-value (OR)
Summer	25	87	28.7%	22.12	≤0.001	0.25	<0.01
Rainy	42	78	35.0%			0.47	<0.01
Winter	52	45	53.6%			1	–^*b*^

^*a*^ See the supplemental material. The chi-square test analysis for summer and rainy season yielded: *χ^2^* = 4.46, *P* = 0.035. The chi-square test analysis for summer and winter yielded: *χ^2^* = 22.09, *P* ≤ 0.001. The chi-square test analysis for rainy season and winter yielded: *χ^2^* = 7.46, *P* ≤ 0.01.

^*b*^ –, not applicable.

Table 4 further contextualizes the seasonal trends by detailing serotype composition across carcass, fecal, and pork samples. In summer, the serotype composition exhibited broad heterogeneity, with multiple serotypes contributing comparably across matrices. This diffuse serotype landscape, highlighted by the co-occurrence of *S.* Weltevreden, *S.* Bovismorbificans, *S.* Rissen, and several minor serotypes, serves as the baseline from which the subsequent seasonal shifts can be interpreted. In contrast, the rainy season represents a directional shift rather than a mere fluctuation. Carcass isolates became concentrated around *S.* Anatum and *S.* Rissen, while fecal and pork samples showed a marked increase in the prevalence of *S.* Rissen relative to summer. This pattern indicates a coordinated reduction in serotype diversity, suggesting an increasing homogenization of contamination sources along the pork production chain. By winter, this process reached its structural peak. Carcass isolates consisted almost exclusively of *S.* Rissen and *S.* enterica ser. 4,5,12:i:-, while fecal isolates were similarly dominated by *S.* Rissen, along with smaller clusters of *S.* Kedougou and *S.* enterica ser. 4,5,12:i:-. Pork samples exhibited the highest concentration of *S.* Rissen in winter (37 isolates), representing the most concentrated serotype profile observed across all seasons. The collapse in diversity across matrices aligns precisely with the statistical signal detected in Table 3, reinforcing winter as the season of greatest serotype convergence.

**TABLE 4:** Serotypes of *Salmonella* in different sample categories (carcass, feces, pork) across seasons^*a*^

Season	Carcass			Feces			Pork		
	Serotype	No.	Rate	Serotype	No.	Rate	Serotype	No.	Rate
Summer	*S.* Weltevreden	6	5.4%	*S.* Weltevreden	21	18.8%	***S.* Rissen**	**16**	**14.3%**
	*S.* Typhimurium	3	2.7%	*S.* Bovismorbificans	14	12.5%	*S.* Weltevreden	11	9.8%
	*S.* Bovismorbificans	2	1.8%	***S.* Rissen**	**9**	**8.1%**	*S.* Anatum	10	8.9%
	*S.* Derby	1	0.9%	*S.* Derby	4	3.6%	*S.* Bovismorbificans	3	2.7%
	*S.* Infantis	1	0.9%	*S.* 4,5,12:i:-	4	3.6%	Cannstatt	1	0.9%
				*S.* Anatum	3	2.7%	*S.* Newport	1	0.9%
				*S.* Kedougou	1	0.9%	No serotype	1	0.9%
Rainy	*S.* Anatum	5	4.2%	***S.* Rissen**	**13**	**10.8%**	***S.* Rissen**	**24**	**20%**
	***S.* Rissen**	**5**	**4.2%**	*S.* Weltevreden	5	4.2%	*S.* Anatum	23	19.2%
	*S.* Fulda	2	1.7%	*S.* Anatum	4	3.3%	*S.* Derby	5	4.2%
	*S.* Bovismorbificans	1	0.8%	*S.* Derby	4	3.3%	*S.* Hvittingfoss	4	3.3%
				*S.* Typhimurium	4	3.3%	*S.* Newport	4	3.3%
				*S.* Stanley	2	1.7%	*S.* 4,5,12:i:-	3	2.5%
				*S.* Wandsworth	1	0.8%	*S.* Wandsworth	3	2.5%
				*S.* 4,5,12:i:-	1	0.8%	*S.* Bovismorbificans	2	1.7%
							Mbandaka	2	1.7%
							*S.* Give	1	0.8%
							*S.* Fulda	1	0.8%
							*S.* Stanley	1	0.8%
Winter	***S.* Rissen**	**2**	**2.1%**	***S.* Rissen**	**13**	**13.4%**	***S.* Rissen**	**37**	**38.1%**
	*S.* 4,5,12:i:-	2	2.1%	*S.* Kedougou	5	5.2%	*S.* Hvittingfoss	7	7.2%
				*S.* 4,5,12:i:-	4	4.1%	*S.* Stanley	5	5.2%
				*S.* Stanley	3	3.1%	*S.* Anatum	4	4.1%
				*S.* Wandsworth	3	3.1%	*S.* 4,12:i:-	3	3.1%
				*S.* Hvittingfoss	1	1%	*S.* Bredeney	1	1%
							*S.* Kedougou	1	1%
							*S.* krefeld	1	1%
							*S.* Newport	1	1%
							*S.* Typhimurium	1	1%
							*S.* Urbana	1	1%
							*S.* Weltevreden	1	1%
							No serotype	1	1%

^*a*^ *S.* Rissen prevalence is based on the total number of isolates, not total samples. The increase observed in winter is mainly due to a decrease in other serovars, not an actual rise in *S.* Rissen itself. This reflects a reduction in serotype diversity rather than a true increase in prevalence. Boldface indicates the serotypes with the highest prevalence in each sample category/season.

When interpreted collectively, Fig. 2 and Tables 3 and 4 depict a coherent seasonal trajectory in which *S.* Rissen transitions from one of several co-dominant serotypes in summer to the overwhelmingly predominant serotype in winter. The rainy season functions as a pivot point marked by rising *S.* Rissen prevalence and contraction of secondary serotypes. Statistical analyses not only corroborate these visual trends but also quantify the strength of the winter shift, revealing significantly increased odds of *S*. Rissen isolation during this period. These findings position winter as a critical phase during which serotype structure becomes tightly consolidated, likely reflecting shifts in environmental persistence, production-chain contamination dynamics, or both. The strong coherence between visual patterns, statistical outputs, and cross-matrix serotype behavior produces a unified framework for understanding temporal fluctuations in *Salmonella* ecology across the pork supply chain.

### Antimicrobial susceptibility testing and PFGE analysis

The multivariable logistic regression was used to quantify independent predictors of antimicrobial resistance while controlling for potential confounding across season, sample matrix, and serotype (Table 5). Season exhibited a significant effect on resistance. After adjustment, isolates obtained during the rainy season were substantially more likely to be antimicrobial-resistant compared with those obtained in winter (reference), with an estimated odds ratio (OR) of 11.9 (*P* = 0.002). In contrast, isolates collected in summer did not differ significantly from winter (OR = 1.5, *P* = 0.53). Sample matrix did not emerge as a statistically significant predictor after adjustment. Relative to carcass isolates (reference), both fecal (OR = 0.004, *P* = 0.341) and pork isolates (OR = 0.008, *P* = 0.406) showed reduced point estimates for resistance, although 95% confidence intervals included unity, indicating limited evidence for matrix-associated differences. Serotype contributed appreciably to variation in resistance. Using *S*. Rissen as the reference category due to its epidemiological predominance, *S*. Bovismorbificans demonstrated significantly lower odds of resistance (OR = 0.2, *P* = 0.037). “Other serotypes” also showed reduced resistance compared with *S*. Rissen (OR = 0.2, *P* = 0.008). Although *S*. Weltevreden and *S*. Anatum yielded OR values below 1 (0.3 and 0.3, respectively), the associations were not statistically significant (*P* = 0.13 and 0.22).

**TABLE 5:** Multivariable logistic regression analysis of predictors of antimicrobial resistance in *Salmonella* isolates

Variable	Independent variable	Resistance	Non-resistance	OR	*P*-value
Season	Summer	97	15	1.5	0.53
	Rainy	118	2	11.9	0.002
	Winter (ref)	79	18	–^*a*^	–
Matrix	Carcass (ref)	30	0	–	–
	Feces	99	20	0.004	0.341
	Pork	165	15	0.008	0.406
Serotype	*S*. Rissen (ref)	114	5	–	–
	*S.* Weltevreden	37	7	0.3	0.13
	*S.* Anatum	44	5	0.3	0.22
	*S.* Bovismorbificans	17	5	0.2	0.037
	Others	82	13	0.2	0.008

^*a*^ –, not applicable.

Collectively, the model indicates that seasonality, particularly during the rainy period, is a major independent determinant of resistance, whereas serotype-level variation accounts for part of the residual heterogeneity in resistance outcomes. These findings reinforce the contributions of both environmental conditions and lineage-specific traits to AMR dynamics in the slaughterhouse–market continuum.

**Fig 3:**
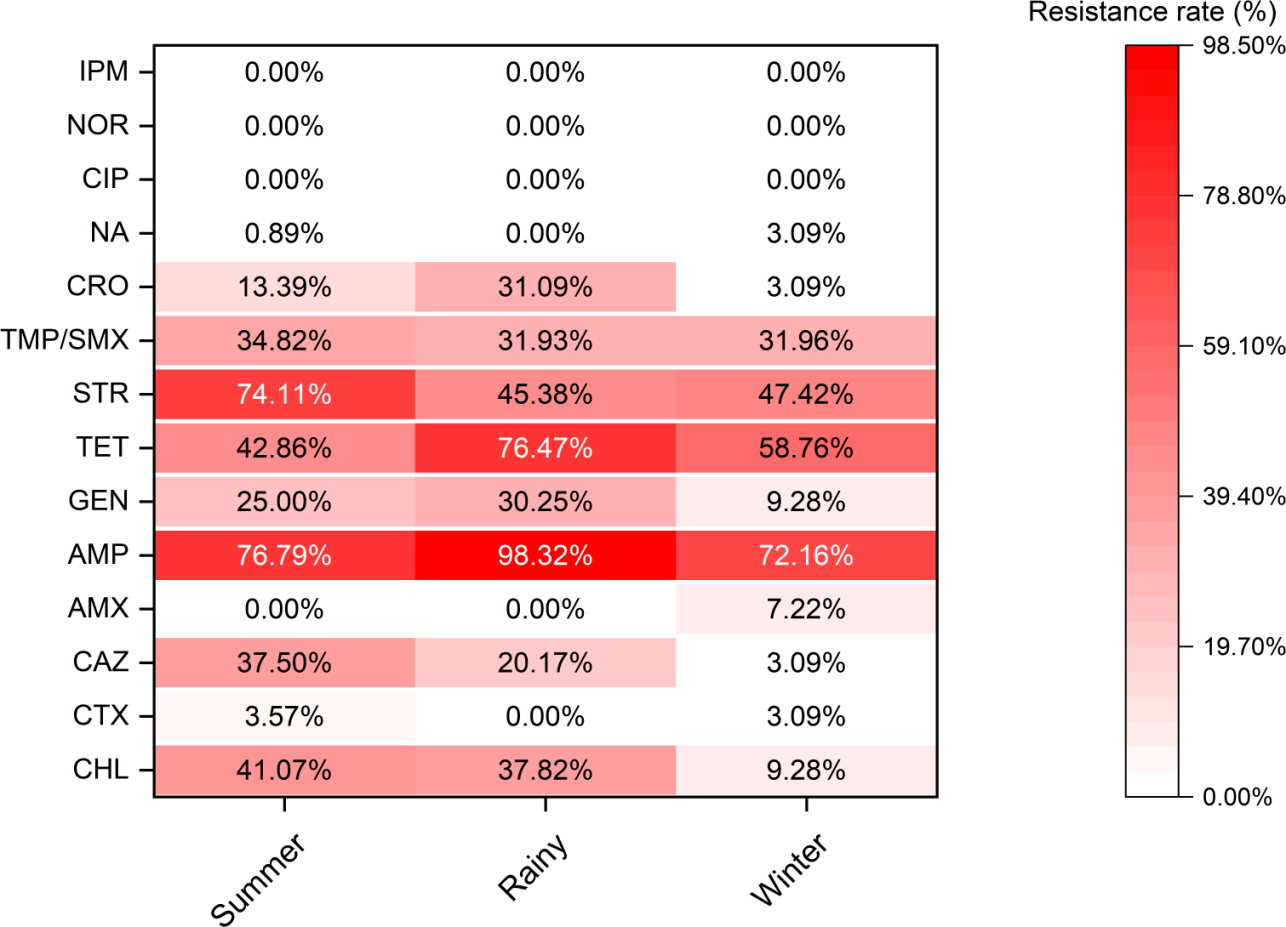
Seasonal distribution of *Salmonella* antimicrobial resistance (AMR) rates. Note: IPM, imipenem; NOR, norfloxacin; CIP, ciprofloxacin; NA, nalidixic acid; CRO, ceftriaxone; TMP/SMX, trimethoprim–sulfamethoxazole; STR, streptomycin; TET, tetracycline; GEN, gentamicin; AMP, ampicillin; AMX, amoxicillin; CAZ, ceftazidime; CTX, cefotaxime; CHL, chloramphenicol.

Seasonal variations in resistance were observed for several antimicrobial classes (Fig. 3). Resistance to AMP was uniformly high, reaching its maximum during the rainy season (98.3%), compared with summer (76.8%) and winter (72.2%). A similar seasonal peak was noted for TET, with resistance rising from 42.9% in summer to 76.5% in the rainy season, before decreasing to 58.8% in winter. STR resistance also showed a moderate seasonal shift, with the highest level detected in summer (74.1%), followed by winter (47.4%) and the rainy season (45.4%). For β-lactam agents, resistance to CAZ and CRO was highest in summer (37.5% and 13.4%, respectively) and declined markedly in winter (both 3.1%). Resistance to CHL remained relatively stable across seasons (37.8%–41.1%). No resistance to NOR, CIP, or amoxicillin was detected in any season. Taken together, the heatmap highlights drug-specific seasonal shifts most prominently for AMP, TET, STR, and TMP/SMX, while indicating that other agents, including third-generation cephalosporins and CHL, displayed more modest or inconsistent seasonal variation.

Distinct serotype-dependent resistance characteristics were observed among carcass isolates across seasons (Table 6). In summer, the most notable finding was the high-frequency MDR phenotype (AMP/TET/STR) in *S.* Weltevreden (83.3%), which contrasted with *S.* Typhimurium, where STR-associated profiles dominated. During the rainy season, *S.* Anatum displayed the broadest resistance spectrum among carcass serotypes, including the CHL/CAZ/AMP combination, whereas *S.* Rissen was the only serotype exhibiting multiple phenotypes within the same season, ranging from single-drug (AMP) to MDR forms (e.g., CHL/CAZ/AMP). In winter, MDR patterns disappeared entirely; both *S.* Rissen and *S.* 4,5,12:i:- were characterized exclusively by APM resistance, indicating a marked shift from multidrug to mono-drug resistance among carcass isolates in the cold season. Overall, only a small subset of serotypes contributed to seasonal AMR variability in carcass samples, with *S.* Weltevreden, *S.* Anatum, and *S.* Rissen representing the major drivers of multidrug resistance during the warm and rainy months.

**TABLE 6:** Serotype-specific antimicrobial resistance (AMR) patterns among *Salmonella* isolates in carcass samples across different seasons^*a*^

Season	Serotype	Patterns	Number of resistance	Rate (%)
Summer	*S.* Weltevreden	AMP/TET/STR	5	83.3%
		AMP/STR	1	16.7%
	*S*. Typhimurium	AMP/TET/STR	1	33.3%
		AMP/STR	2	66.7%
	*S*. Bovismorbificans	STR	2	100.0%
	*S*. Infantis	STR	1	100.0%
	*S.* Derby	STR	1	100.0%
Rainy	*S.* Anatum	CHI/CAZ/AMP	3	60.0%
		AMP/TET	2	40.0%
	*S*. Bovismorbificans	AMP/TET	1	100.0%
	*S.* Fulda	CHI/CAZ/AMP	2	100.0%
	***S*. Rissen**	**CHI/CAZ/APM**	3	60.0%
		**AMP**	1	20.0%
		**AMP/TET**	1	20.0%
Winter	***S*. Rissen**	**APM**	2	50.0%
	*S.* 4,5,12:i:-	APM	2	50.0%

^*a*^ The abbreviation S denotes isolates that were fully susceptible (non-resistant) to all antimicrobials tested in the panel. Each resistance pattern corresponds to the unique, full resistance profile of one isolate. Thus, subsets of larger patterns were not duplicated, and no isolate contributed to more than one pattern. Boldface indicates the serotypes with the highest prevalence in each sample category/season.

Marked serotype-dependent differences in fecal AMR patterns were observed across seasons (Table 7). In summer, *S.* Rissen and *S.* Weltevreden were the primary contributors to MDR, with *S.* Rissen showing AMP/TET/STR/TMP/SMX-based profiles and *S.* Weltevreden exhibiting extended combinations involving cephalosporins. Several minor serotypes (e.g., *S*. Kedougou, *S*. 4,5,12:i:-) also displayed MDR, indicating high pattern diversity during this period. In the rainy season, MDR profiles became concentrated within fewer serotypes, with *S.* Rissen maintaining the widest spectrum, including combinations with CRO. Other serotypes largely presented simplified AMP/TET-based resistance, reflecting a narrowing of MDR distribution. By winter, MDR phenotypes were substantially reduced, with only *S.* 4,5,12:i:- and *S.* Rissen retaining multidrug resistance, while most remaining serotypes exhibited only single- or dual-drug profiles. Overall, fecal isolates showed strong seasonal shifts in MDR, driven predominantly by *S.* Rissen, with the most complex patterns occurring in summer and progressively simplifying toward winter.

**TABLE 7:** Serotype-specific antimicrobial resistance (AMR) patterns among *Salmonella* isolates in feces samples across different seasons^*a*^

Season	Serotype	Patterns	Number of resistance	Rate (%)
Summer	*S.* Weltevreden	AMP/STR	1	4.8%
		AMP/STR/TMP/SMX	1	4.8%
		CHI/CAZ/AMP/GEN/TET/STR/TMP/SMX/CRO	2	9.5%
		STR	4	19.0%
		CHI/CAZ/AMP/GEN/TET/STR	7	33.3%
		S	6	28.6%
	***S*. Rissen**	**AMP/TET/STR/TMP/SMX**	2	22.2%
		**S**	1	11.1%
		**AMP/STR**	6	66.7%
	*S.* Kedougou	AMP/STR/TMP/SMX	1	100.0%
	*S.* Derby	STR	2	50.0%
		CHI/CAZ/AMP/GEN/TET/STR/TMP/SMX/CRO	1	25.0%
		S	1	25.0%
	*S.* Bovismorbificans	STR	1	7.1%
		CHI/CAZ/AMP/GEN/TET/STR/TMP/SMX/CRO	2	14.3%
		CHI/CAZ/AMP/GEN/TET/STR	6	42.9%
		S	5	35.7%
	*S.* Anatum	CHI/CAZ/AMP/GEN/TET/STR/TMP/SMX/CRO	1	33.3%
		S	2	66.7%
	*S.* 4,5,12:i:-	AMP/STR	2	50.0%
		AMP/STR/TMP/SMX	2	50.0%
Rainy	*S.* Weltevreden	CHI/CAZ/AMP/GEN/TET/STR/TMP/SMX/CRO	1	20.0%
		CHI/AMP/GEN/TET/STR/TMP/SMX/CRO	1	20.0%
		AMP/TET	3	60.0%
	*S.* Wandsworth	AMP/TET	1	100.0%
	*S*. Typhimurium	AMP/TET	3	75.0%
		AMP	1	25.0%
	*S.* Stanley	AMP/TET	2	100.0%
	***S*. Rissen**	**AMP**	9	69.2%
		**AMP/TET**	2	15.4%
		**CHI/CAZ/AMP/GEN/TET/STR/TMP/SMX/CRO**	2	15.4%
	*S.* 4,5,12:i:-	AMP	1	100.0%
	*S.* Derby	AMP	1	25.0%
		AMP/TET	3	75.0%
	*S.* Anatum	AMP	1	25.0%
		AMP/TET	3	75.0%
Winter	*S.* 4,5,12:i:-	CHI/AMP/TET/STR	1	25.0%
		AMP/TET/STR	2	50.0%
		S	1	25.0%
	*S.* Stanley	AMP	1	33.3%
		GEN	1	33.3%
		S	1	33.3%
	*S.* Hvittingfoss	S	1	100.0%
	*S.* Wandsworth	TET	3	100.0%
	*S.* Kedougou	GEN	1	20.0%
		AMP/TMP/SMX	1	20.0%
		AMP/STR/TMP/SMX	1	20.0%
		S	2	40.0%
	***S*. Rissen**	**AMX/AMP**	1	7.7%
		**CHI/CTX/CAZ/AMP/TET/STR/NA/TMP/SMX/CRO**	1	7.7%
		**CHI/CTX/CAZ/AMP/TET/STR/CRO**	1	7.7%
		**AMP/STR**	1	7.7%
		**AMP**	1	7.7%
		**AMP/TET/TMP/SMX**	4	30.8%
		**AMP/TET/STR/TMP/SMX**	4	30.8%

^*a*^ The abbreviation S denotes isolates that were fully susceptible (non-resistant) to all antimicrobials tested in the panel. Each resistance pattern corresponds to the unique, full resistance profile of one isolate. Thus, subsets of larger patterns were not duplicated, and no isolate contributed to more than one pattern. Boldface indicates the serotypes with the highest prevalence in each sample category/season.

Pronounced serotype-specific differences in antimicrobial resistance patterns were observed among pork isolates across seasons (Table 8). In summer, *S.* Rissen exhibited the widest MDR spectrum, including complex profiles incorporating cephalosporins (e.g., CHL/CAZ/AMP/GEN/TET/STR/TMP/SMX/CRO). *S*. Weltevreden also contributed notable MDR combinations, whereas most other serotypes displayed only limited patterns. During the rainy season, resistance profiles expanded to additional serotypes, with *S.* Anatum, *S.* Bovismorbificans, and several low-prevalence serotypes expressing diverse MDR phenotypes. *S.* Rissen remained the principal MDR contributor, presenting multiple extended patterns containing both β-lactams and TMP/SMX, indicating a broader dissemination of MDR phenotypes in pork during this period. By winter, MDR diversity decreased markedly. Although S. Rissen continued to exhibit multiple MDR combinations, most other serotypes, including *S*. Anatum, *S.* Bredeney, S. Kedougou, and *S*. 4,5,12:i:-, displayed either simplified or infrequent resistance profiles. Only a small subset of isolates retained extended MDR phenotypes, demonstrating a seasonal contraction of AMR complexity in pork samples. Overall, pork isolates exhibited the highest diversity of MDR profiles among all sample types, with *S.* Rissen consistently serving as the major driver of multiclass resistance and the most complex resistance combinations occurring during the summer and rainy seasons.

**TABLE 8:** Serotype-specific antimicrobial resistance (AMR) patterns among *Salmonella* isolates in pork samples across different seasons^*a*^

Season	Serotype	Patterns	Number of resistance	Rate (%)
Summer	*S.* Weltevreden	CHI/AMP/STR/TMP/SMX	2	18.2%
		AMP/TET/STR/TMP/SMX	7	63.6%
		CHI/CZA/AMP	2	18.2%
	***S*. Rissen**	**CHI/AMP/STR/TMP/SMX**	1	6.3%
		**CHI/CAZ/AMP/GEN/TET/STR/TMP/SMX/CRO**	5	31.3%
		**CHI/CTX/CAZ/AMP/GEN/TET/STR/TMP/SMX/CRO**	4	25.0%
		**AMP/TET/STR/TMP/SMX**	1	6.3%
		**AMP/TET/STR**	1	6.3%
		**CHI/CAZ/AMP**	4	25.0%
	*S.* Cannstatt	CHI/APM/STR/TMP/SMX	1	100.0%
	*S.* Newport	APM/TET/STR/TMP/SMX	1	100.0%
	*S*. Bovismorbificans	AMP/STR/TMP/SMX	3	100.0%
	*S.* Anatum	CHI/CAZ/AMP	8	80.0%
		AMP/TET/STR/TMP/SMX	2	20.0%
	No serotype	AMP/STR/NA	1	100.0%
Rainy	*S.* Anatum	CHI/CAZ/AMP/GEN/TET/STR/TMP/SMX/CRO	4	17.4%
		CHI/AMP/GEN/TET/STR/TMP/SMX/CRO	12	33.3%
		AMP/TET	4	17.4%
		TMP/SMX	1	4.3%
		S	2	8.7%
	*S*. Bovismorbificans	AMP/TET/STR	2	100.0%
	*S.* Derby	AMP/TET/STR	1	20.0%
		CHI/CAZ/AMP/GEN/STR/TMP/SMX/CRO	1	20.0%
		AMP/STR	3	60.0%
	*S.* 4,5,12:i:-	AMP/TET/STR	2	66.7%
		CHI/APM/GEN/TET/STR/TMP/SMX/CRO	1	33.3%
	*S.* Give	CHI/CAZ/AMP/GEN/TET/STR/TMP/SMX/CRO	1	100.0%
	*S.* Fulda	CHI/AMP/GEN/TET/STR/TMP/SMX/CRO	1	100.0%
	*S*. Hvittingfoss	CHI/CAZ/AMP/GEN/TET/STR/TMP/SMX/CRO	3	75.0%
		CHI/AMP/GEN/TET/STR/TMP/SMX/CRO	1	25.0%
	*S*. Mbandaka	CHI/AMP/GEN/TET/STR/TMP/SMX/CRO	2	100.0%
	*S*. Newport	AMP/TET/STR	1	25.0%
		AMP/TET	1	25.0%
		CHI/CAZ/AMP/GEN/TET/STR/TMP/SMX/CRO	2	50.0%
	***S*. Rissen**	**CHI/AMP/GEN/TET/STR/CRO**	1	4.2%
		**CHI/AMP/TET/STR/TMP/SMX/CRO**	1	4.2%
		**CHI/CAZ/AMP/GEN/TET/STR/TMP/SMX/CRO**	1	4.2%
		**AMP/TET**	9	37.5%
		**AMP/TET/STR**	10	41.7%
		**AMP/TET/TMP/SMX**	1	4.2%
		**S**	1	4.2%
	*S.* Stanley	APM/TET	1	100.0%
	*S.* Wandsworth	CHI/AMP/GEN/TET/STR/TMP/SMX/CRO	1	33.3%
		CHI/CZA/AMP/GEN/TET/STR/TMP/SMX/CRO	1	33.3%
		AMP/TET/STR	1	33.3%
Winter	*S.* Anatum	STR	1	25.0%
		AMP	1	25.0%
		S	2	50.0%
	*S*. Bredeney	TET/STR/TMP/SMX	1	100.0%
	No serotype	AMP/TET/STR	1	100.0%
	*S.* Hvittingfoss	S	6	85.7%
		AMP/TET/STR/TMP/SMX	1	14.3%
	*S.* 4,5,12:i:-	AMP/TET/STR/TMP/SMX	1	33.3%
		AMX/AMP/TET/STR/TMP/SMX	1	33.3%
		CHI/AMP/GEN/TET/STR	1	33.3%
	*S.* Kedougou	CHI/AMP/GEN/TET/STR	1	100.0%
	*S.* krefeld	AMP/TET/STR	1	100.0%
	*S.* Newport	AMP/GEN	1	100.0%
	*S.* Weltevreden	S	1	100.0%
	*S*. Urbana	AMP/TET/STR	1	100.0%
	*S.* Stanley	CHI/CTX/CAZ/AMX/AMP/GEN/TET/STR/NA/TMP/ SMX/CRO	1	20.0%
		AMP/TET/STR/TMP/SMX/CRO	1	20.0%
		CHI/AMPGEN//TET/STR	1	20.0%
		AMP	1	20.0%
		S	1	20.0%
	*S*. Typhimurium	AMP/TET/STR	1	100.0%
	***S*. Rissen**	**CHI/AMP/GEN/TET/STR**	2	5.4%
		**AMP/TET/STR/NA**	1	2.7%
		**AMP/TET/STR/TMP/SMX**	7	18.9%
		**AMP/STR/TMP/SMX**	2	5.4%
		**AMP/TET/STR**	6	16.2%
		**TET/STR**	2	5.4%
		**AMP/STR**	1	2.7%
		**AMP/TET/TMP/SMX**	6	16.2%
		**AMP/TET**	4	10.8%
		**AMP**	3	8.1%
		**S**	3	8.1%

^*a*^ Each resistance pattern represents the full resistance profile of a single isolate. Each isolate was counted only once, and no subsets of larger combinations were included. Thus, all listed resistance patterns are mutually exclusive and do not overlap. Boldface indicates the serotypes with the highest prevalence in each sample category/season.

Across all sample types, pronounced heterogeneity was observed in both the distribution and complexity of antimicrobial resistance patterns. Carcass isolates displayed the narrowest resistance spectra, with most serotypes exhibiting only one or two single- or dual-class resistance profiles and limited seasonal fluctuation. In contrast, fecal isolates demonstrated substantially broader and more diverse resistance combinations, including multiple multiclass patterns enriched in ampicillin, tetracycline, streptomycin, and TMP/SMX, particularly among *S*. Weltevreden and *S.* Anatum during the rainy season. Pork isolates exhibited the highest overall MDR diversity. *S.* Rissen consistently emerged as the dominant contributor to multiclass resistance across all seasons, generating the most complex profiles—including combinations of β-lactams, aminoglycosides, tetracycline, and TMP/SMX—and reaching peak diversity during the summer and rainy periods. Several additional serotypes contributed sporadic but extended MDR patterns in pork samples, while winter months showed a contraction in both the number of affected serotypes and the complexity of resistance profiles. Together, these findings indicate a gradient of increasing AMR complexity from carcass to feces to pork, with pork acting as the primary reservoir for MDR phenotypes within the production chain, and *S*. Rissen representing the principal serotype driving multiclass resistance across matrices and seasons.

**Fig 4:**
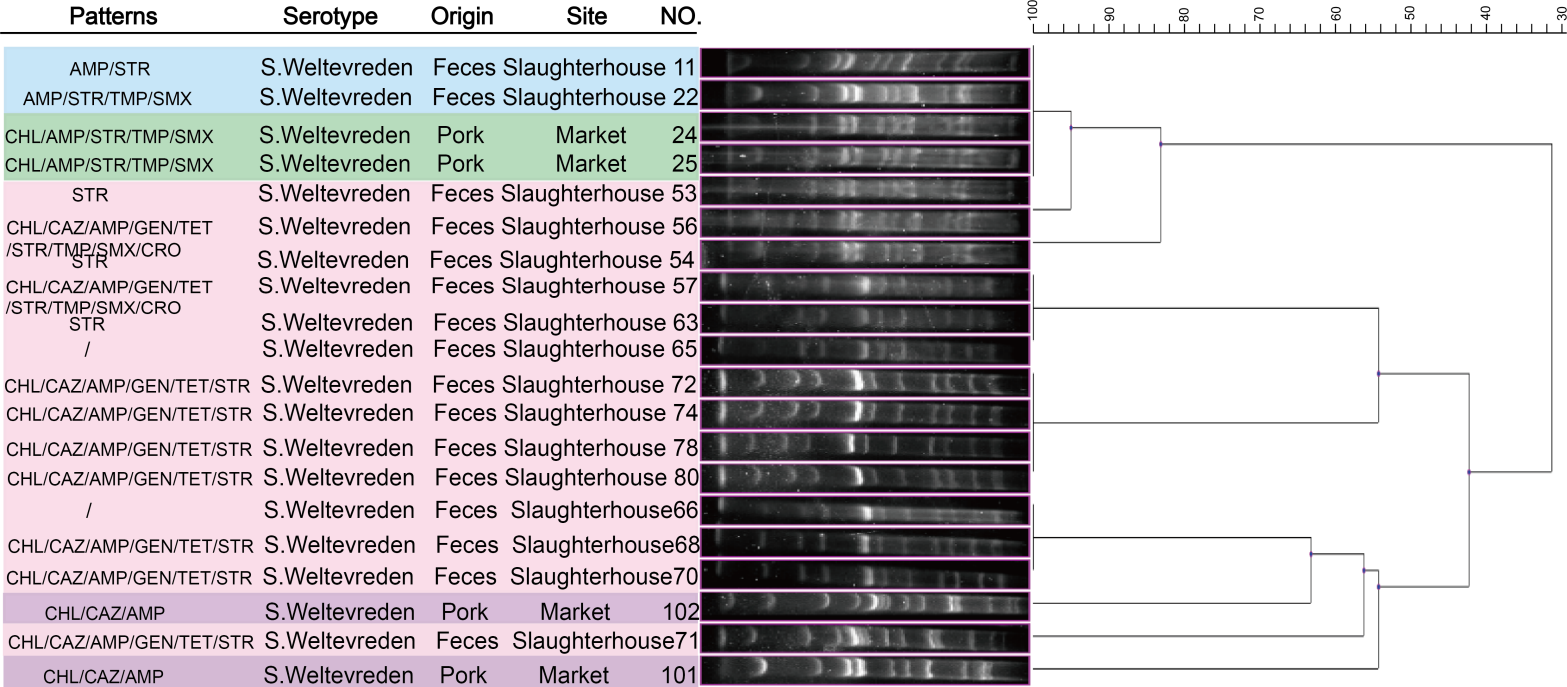
Pulsed-field gel electrophoresis clustering analysis of *S.* Weltevreden serotype in summer. The left side shows the resistance pattern (Pattern), sample serotype (Serotype), sample origin (Origin), sampling site (Site), and strain number (NO.). The middle shows the PFGE bands for each strain. The right side shows the phylogenetic tree, with the top scale indicating similarity (%). CHL, chloramphenicol; CAZ, cefotaxime; CRO, ceftriaxone; AMP, ampicillin; GEN, gentamicin; TET, tetracycline; STR, streptomycin; TMP/SMX, trimethoprim/sulfamethoxazole; NA, naphthoquinone. “/” indicates sensitivity to all tested antibiotics. Slaughterhouses from Udon Thani Province display a blue background. Market 1 from Udon Thani Province displays a green background. Slaughterhouses from Khon Kaen Province display a pink background. Market 2 from Khon Kaen Province displays a purple background.

An analysis was conducted on 20 strains of *S.* Weltevreden that were dominant during the summer (Fig. 4), primarily sourced from slaughterhouse manure (16/20), with the remainder from market pork (4/20). PFGE band patterns showed significant polymorphism, with similarity ranges of approximately 55%–100%. Strains from different sampling sites and sources were interspersed on the clustering tree, with no obvious source-based clustering observed. Antimicrobial resistance profiles varied significantly. High-level MDR strains (CHL/CAZ/AMP/GEN/TET/STR/TMP/SMX/CRO) were primarily concentrated in isolates from slaughterhouse manure and formed closely related clusters with similar bands on the clustering tree. Low-resistance strains (e.g., STR, AMP/STR, AMP/STR/TMP/SMX) and strains fully sensitive to all tested antibiotics (“/”) were distributed across different branches. Two strains in market pork exhibited identical resistance patterns (CHL/AMP/STR/TMP/SMX) and highly similar banding patterns, suggesting the possible circulation of the same clone in the market.

**Fig 5:**
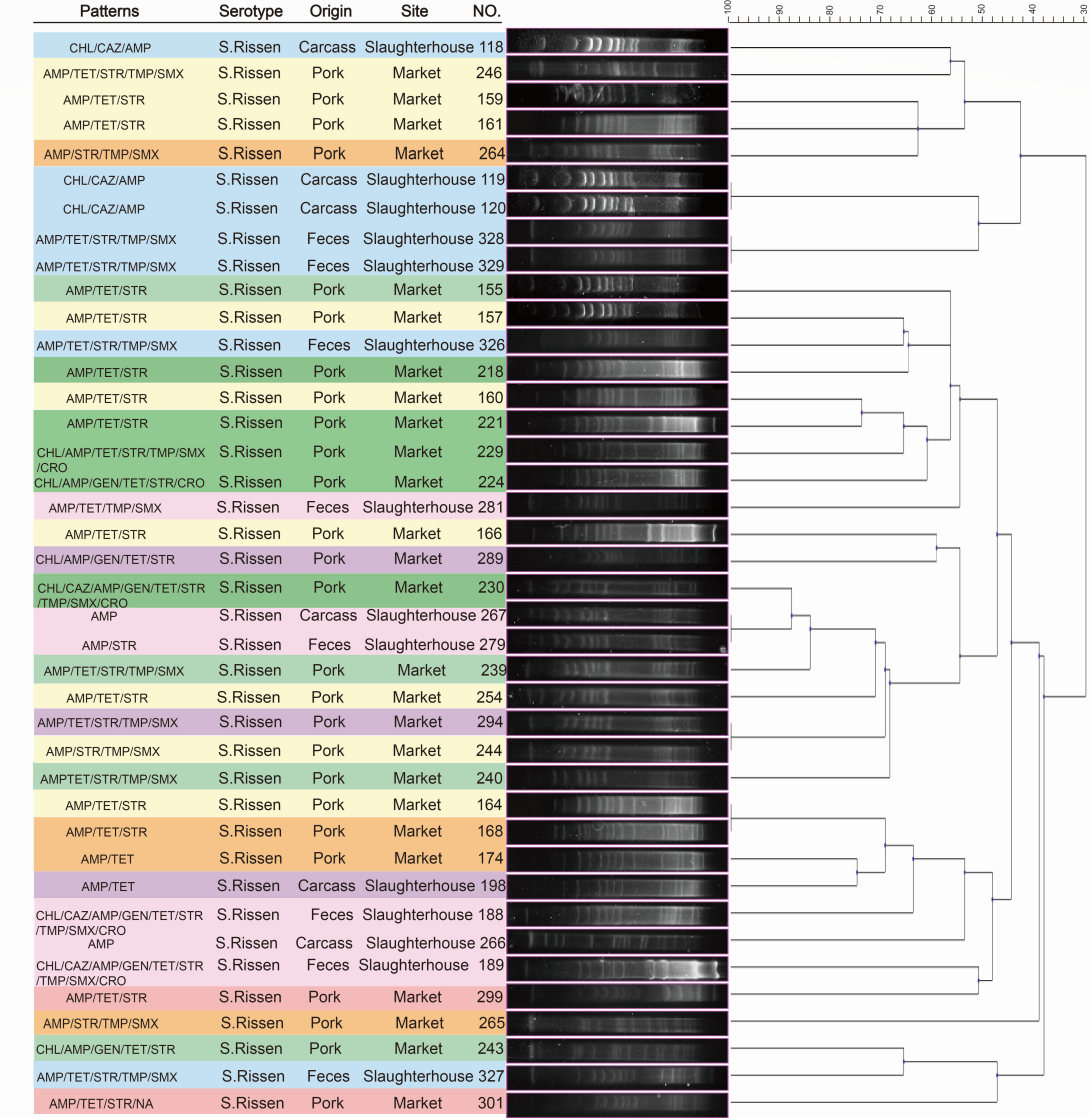
Pulsed-field gel electrophoresis clustering analysis of *S.* Rissen serotype in rainy and winter. Strains numbered 1–20 were isolated and identified during the rainy season. Strains numbered 21–40 were isolated and identified during the winter season. The left side listed the resistance pattern (Pattern), sample serotype (Serotype), sample origin (Origin), sampling site (Site), and strain number (NO.). The middle listed the PFGE bands for each strain. The right side shows the phylogenetic tree, with the top scale indicating similarity (%). CHL, chloramphenicol; CAZ, cefotaxime; CRO, ceftriaxone; AMP, ampicillin; GEN, gentamicin; TET, tetracycline; STR, streptomycin; TMP/SMX, trimethoprim/sulfamethoxazole; NA: naphthoquinone. “/” indicates sensitivity to all tested antibiotics. Slaughterhouses from Udon Thani Province display a light pink background. Market 1 from Udon Thani Province displays a purple background. Market 2 from Udon Thani Province displays a bright pink background. Market 3 from Udon Thani Province displays a bright green background. Slaughterhouses from Khon Kaen Province display a blue background. Market 1 from Khon Kaen Province displays a light green background. Market 2 from Khon Kaen Province displays a yellow background. Market 3 from Khon Kaen Province displays an orange background.

Another analysis was conducted on 40 *S.* Rissen strains that dominated in terms of quantity during the rainy season and winter (Fig. 5, rainy season strains numbered 1–20; winter strains numbered 21–40). Among these, the rainy season strains were primarily sourced from market pork (13/20), with 4/20 from slaughterhouse carcasses and 3/20 from manure. Consistently, the winter strains were primarily sourced from market pork (13/20), with 5/20 from slaughterhouse manure and 2/20 from carcasses. PFGE band polymorphism was high, with similarity ranging from approximately 50% to 100%. Strains from different seasons did not form independent branches, and similar types were interspersed between the rainy season and winter, indicating the presence of closely related clonal types with cross-seasonal transmission. Antimicrobial-resistant strains were dominated by AMP/TET/STR (ATS) and its extended form AMP/TET/STR/TMP/SMX, which were widely present in both seasons. Additionally, multiple ATS-type strains exhibit highly similar bands and were distributed across different sources and sampling sites, including both slaughterhouses and markets. Some strains carry third-generation cephalosporin resistance (e.g., CHL/CAZ/AMP, CHL/AMP/GEN/TET/STR/CRO, CHL/CAZ/AMP/GEN/TET/STR/TMP/SMX/CRO), and these MDR strains were distributed in different matrices in slaughterhouses and markets. A few strains exhibited NA in their resistance profiles (e.g., AMP/TET/STR/NA).

Whether from *S.* Weltevreden or *S.* Rissen, strains from different sources (slaughterhouses/markets) and sample types (feces/carcasses/pork) did not form distinct source- or season-specific branches in PFGE clustering, suggesting that similar clones may persistently circulate between slaughter processing chains and markets. *S.* Weltevreden was dominated by MDR clusters in summer, while *S.* Rissen exhibited ATS-dominant patterns during the rainy season and winter, accompanied by the presence of third-generation cephalosporin MDR subclusters. The lack of clear segregation between market and slaughterhouse isolates in the PFGE analysis highlights the complex, interrelated dynamics between these two environments in the circulation of resistant strains.

### Phylogenetic analysis

**Fig 6:**
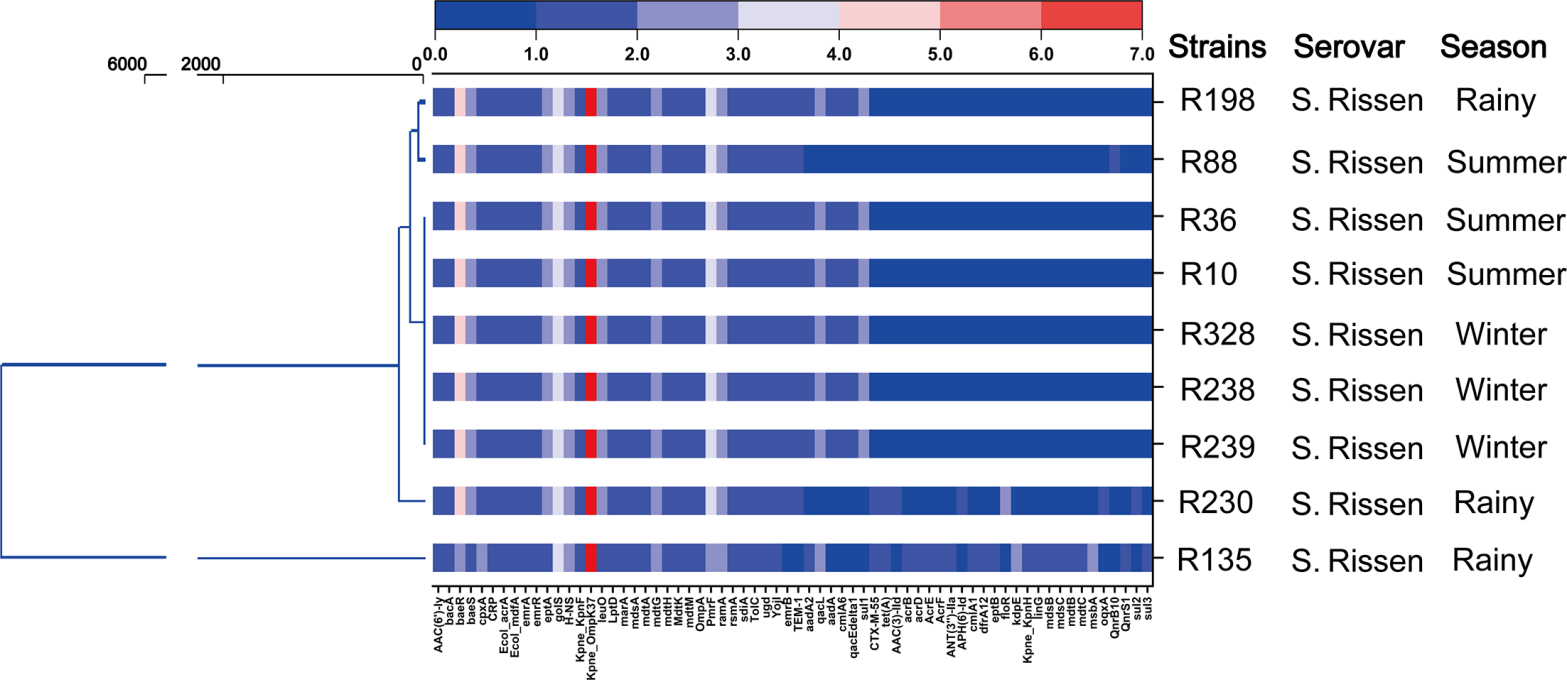
Maximum-likelihood genetics of *Salmonella* isolates, heat map showing the total number of resistance genes across the entire genome. Strain 10 originated from a slaughterhouse in Udon Thani Province. Strains 36 and 198 originated from Market 1 in Udon Thani Province. Strains 88, 238, and 239 originated from Market 1 in Khon Kaen Province. Strains 218 and 230 originated from Market 3 in Udon Thani Province. Strain 328 originated from a slaughterhouse in Khon Kaen Province.

The maximum-likelihood phylogenetic tree revealed that the nine *Salmonella enterica* serotype Rissen isolates clustered into two distinct lineages (Fig. 6). One lineage comprised isolates obtained during the rainy (198) and summer seasons (88, 36, 10), while the second lineage included three winter isolates (328, 238, 239) together with two rainy season isolates (230, 218). Notably, the winter isolates formed a closely related cluster, which is associated with high genetic similarity, whereas the rainy and summer season isolates displayed comparatively greater diversity. The strain origins from slaughterhouses and markets were closely linked to the genetic clustering. Strain 10, originating from a slaughterhouse in Udon Thani Province, clustered separately from the market samples, suggesting genetic divergence related to the source of contamination. Meanwhile, Strains 36 and 198, obtained from Market 1 in Udon Thani Province, grouped closely with Strains 88, 238, and 239, which were obtained from Market 1 in Khon Kaen Province. This suggests a possible shared contamination route between these two markets or similar environmental conditions within the markets. Furthermore, Strains 218 and 230, originating from Market 3 in Udon Thani Province, formed a distinct cluster, further supporting the idea that market-specific genetic variations exist. The resistance gene heat map revealed that all isolates shared a highly conserved multidrug resistance profile. Core determinants included efflux pump systems (*acrAB-tolC*, *emrAB*, *mdtABC*, *oqxA*), porin-related genes (*ompA*, *LptD*, *Kpne_OmpK37*), and global transcriptional regulators (*marA*, *ramA*, *sdiA*, *baeSR*, *cpxAR*). In addition, acquired resistance genes were consistently detected across isolates, such as β-lactamases (*blaTEM-1*, *blaCTX-M-55*), aminoglycoside resistance genes (*aadA*, *aac(6′)-Iy*, *aac(3)-IId*), phenicol resistance genes (*cmlA6*, *floR*), sulfonamide resistance genes (*sul1–3*), trimethoprim resistance gene (*dfrA12*), and plasmid-mediated quinolone resistance genes (*qnrB10*, *qnrS1*). While the overall resistance architecture was highly similar across all isolates, Strains 230 and 218 (rainy season) were associated with slightly higher abundance of efflux- and PMQR-related determinants.

Collectively, these findings suggested that *S.* Rissen isolates circulating in different seasons maintain a conserved multidrug resistance (MDR) backbone with only limited seasonal variation and that contamination sources (e.g., markets vs slaughterhouses) may play a role in the genetic divergence observed.

## DISCUSSION

This study provided a comprehensive, season-stratified assessment of *Salmonella* contamination, antimicrobial resistance (AMR), serotype diversity, and genetic relatedness across multiple sampling locations including farm-linked slaughterhouses and retail markets in northeastern Thailand. The analysis revealed pronounced seasonal variation and location-specific differences, with retail environments showing disproportionately higher contamination compared with upstream processing stages. These findings highlight both persistent clonal circulation and dynamic shifts driven by seasonal and infrastructure-related factors, underscoring their implications for regional food safety and AMR management.

In this study, *Salmonella* prevalence displayed marked seasonal variation, with winter consistently showing the lowest contamination across all sample matrices. Carcass positivity decreased to 4.0% in winter, compared with 11.3% in summer and 13.0% during the rainy season. Fecal samples showed a similar pattern, increasing from 29.0% in winter to 50.5% in summer and 34.0% during the rainy season. Retail pork exhibited the highest overall contamination, reaching 75.4% in summer, 75.3% in the rainy season, and 54.7% in winter, underscoring retail markets as a critical point of consumer exposure within the slaughter-to-market continuum.

The lower prevalence observed in winter may reflect cooler and drier conditions that suppress environmental multiplication of *Salmonella* and reduce opportunities for cross-contamination during slaughter, transport, and retail handling. Although low temperatures do not necessarily reduce *Salmonella* survival, the reduced bacterial growth rates under cooler conditions likely limit amplification along the production chain, resulting in lower detectable loads in winter. In contrast, elevated temperature and humidity during summer and the rainy season are associated with enhanced bacterial persistence on surfaces, in water, and on equipment, facilitating transmission and accumulation along the supply chain (16). These seasonal effects were particularly pronounced at downstream sampling locations, where retail pork demonstrated substantially higher contamination than carcass or fecal samples, suggesting that environmental conditions interact with market-level infrastructure such as limited cold-chain capacity and prolonged exposure to warm, humid environments, to exacerbate bacterial accumulation. Similar seasonal peaks during warmer, more humid months have been reported in China and Vietnam (17, 18), while European surveillance also identifies summer as the peak period for *Salmonella* outbreaks (19). U.S. retail meat monitoring further supports the influence of climatic factors on pathogen prevalence (20). Our finding that market pork exhibited the highest contamination levels (≈75%) closely aligns with studies across Southeast Asia, where traditional wet markets frequently report >60–70% *Salmonella* positivity due to limited cold-chain infrastructure, high-frequency surface contact, and persistent environmental contamination (21, 22). The “low carcass, high fecal” pattern observed in slaughterhouses is also consistent with Teerarat study (8), reinforcing that fecal shedding is the primary source of contamination, while carcass-level detection depends strongly on hygienic practices during evisceration and washing. Moreover, the magnitude of contamination across sample locations in this study suggests that regional infrastructure differences, particularly between controlled slaughterhouse environments and less regulated retail markets, play an important role in shaping overall prevalence. Seasonal temperature and humidity appear to amplify these structural vulnerabilities, leading to disproportionately higher contamination at the retail stage in northeastern Thailand.

Serotype *S.* Rissen is widely recognized as the predominant pig-associated serotype in Southeast Asia (5); however, our data demonstrate that its dominance fluctuated seasonally. In this study, *S.* Rissen accounted for >70% of winter isolates but fell to <40% during summer, indicating a seasonal shift in serotype ecology. Minor serotypes also showed pronounced temporal variation: *S*. Weltevreden and *S.* Bovismorbificans peaked during the hot and rainy seasons, while *S.* Hvittingfoss and *S*. Kedougou were more prominent during winter. Such shifts are consistent with reports linking *Salmonella* seasonality to changes in environmental reservoirs, rainfall, water contamination, and wildlife activity (23, 24). Moreover, the enrichment of *S.* Weltevreden in fecal samples and *S.* Rissen in pork suggests distinct contamination pathways and survival traits during slaughter and retail handling (25). These findings underscore the importance of season-specific control strategies rather than assuming stable serotype distributions year-round.

The universal absence of resistance to imipenem (IPM), norfloxacin (NOR), and ciprofloxacin (CIP) across all seasons was encouraging and aligns with regional surveillance reports indicating the retained efficacy of these critical drugs (26). In contrast, resistance to ampicillin (AMP), tetracycline (TET), and streptomycin (STR) was consistently high, particularly the AMS (AMP/TET/STR, ATS) pattern, reflecting entrenched resistance likely driven by antibiotic use in pig production (27). Seasonal variations in resistance rates for CAZ, CRO, GEN, and CHL—particularly elevated in summer and the rainy season—may be explained by shifts in dominant serotypes or emerging clonal lineages with expanded resistance profiles. Diversity of MDR pattern (≥5 antibiotic classes) was more frequent in summer and the rainy season, whereas winter isolates often had simpler resistance signatures. This pattern corresponds with serotype shifts: summer and rainy seasons featured more *S*. Weltevreden and *S.* Bovismorbificans, which in this study were often associated with high-level MDR. In contrast, *S.* Rissen, dominant in winter, more commonly exhibited the ATS phenotype with fewer complex resistance combinations (28). The detection of extended-spectrum cephalosporin resistance across seasons (e.g., CAZ, CRO resistance) is particularly concerning given their use as front-line therapy for invasive salmonellosis in humans (29).

PFGE analysis revealed no clear clustering by season, source, or sample type for either *S.* Weltevreden or *S.* Rissen. This suggests the persistent circulation of certain clones across slaughterhouses and retail environments year-round. High-level MDR *S.* Weltevreden clusters (e.g., CHL/CAZ/AMP/GEN/TET/STR/TMP/SMX/CRO) were primarily associated with slaughterhouse feces in summer, implying upstream amplification at the farm–slaughter interface. In contrast, *S.* Rissen clones, dominated by ATS and ATS + TMP/SMX patterns, were detected across carcasses, feces, and pork during both the rainy and winter seasons. The presence of third-generation cephalosporin-resistant *S.* Rissen subclones across multiple matrices indicates a high degree of environmental persistence and dissemination throughout the supply chain. In this study, we employed a random selection strategy for choosing isolates for further investigation. This approach was deliberately chosen to ensure the representativeness of isolates from each season and sample type (carcass, feces, and pork) without introducing selection bias. By selecting isolates randomly, we aimed to capture a broad spectrum of genetic diversity and to ensure that the results accurately reflect the epidemiological patterns observed across the entire pork supply chain in northeastern Thailand. This method helped avoid any skew in the analysis and ensured a comprehensive view of *S.* Rissen and *S*. Weltevreden circulation and antimicrobial resistance (AMR) profiles.

The phylogenetic analysis demonstrated that *Salmonella enterica* serotype Rissen isolates recovered from different seasons in northeastern Thailand exhibited a high degree of genetic similarity, forming two major lineages with only limited seasonal clustering. In particular, the three winter isolates grouped tightly together, whereas isolates from the rainy and summer seasons were more genetically diverse. These findings suggested that the clonal dissemination of *S.* Rissen may occur more frequently during certain seasons, consistent with previous reports highlighting the clonal spread of specific *Salmonella* serotypes in both animal and food production environments (9). Notably, isolates from different sources, such as slaughterhouses and markets, exhibited genetic segregation, suggesting that the contamination source may influence genetic diversity. In particular, isolates from slaughterhouses tended to form distinct clusters compared to those from markets, possibly due to different environmental conditions or contamination dynamics at these sources. This separation may also reflect the differing ecological pressures on *S.* Rissen as it moves through the pork supply chain, from slaughterhouse to market. The resistance gene profiles further supported the observation of clonal stability. All isolates carried a highly conserved MDR backbone dominated by efflux pump systems, porin-associated determinants, and global transcriptional regulators, together with a broad range of horizontally acquired resistance genes (30). The universal detection of extended-spectrum β-lactamase (ESBL) genes such as *blaCTX-M-55*, together with plasmid-mediated quinolone resistance determinants (*qnrB10, qnrS1*), highlights the clinical relevance of these isolates, as they may substantially compromise the effectiveness of critically important antimicrobials. Importantly, the co-existence of MDR mechanisms, including efflux-mediated tolerance and plasmid-encoded determinants (31, 32), may contribute to the persistence and adaptability of *S.* Rissen in different ecological niches. Although the overall resistance gene profiles were highly conserved, minor seasonal variations were observed. Specifically, rainy season isolates (230 and 218) exhibited a slightly higher abundance of efflux- and PMQR-associated genes compared with other isolates. Such variations may be associated with seasonal differences in selective pressures, such as fluctuations in antimicrobial usage patterns, environmental conditions, or transmission routes along the farm-to-market chain. The variation between market and slaughterhouse isolates could reflect different contamination dynamics, with markets potentially contributing to a higher diversity of *S.* Rissen strains compared to more controlled environments like slaughterhouses. Nevertheless, the strong conservation of resistance determinants across all isolates is associated with *S.* Rissen, which is primarily disseminated as a stable, MDR clone in this region, rather than evolving rapidly in response to seasonal factors or differing contamination sources.

Overall, this study highlights the dual pattern of *Salmonella* circulation in northeastern Thailand, characterized by the persistent dissemination of a stable, MDR *S*. Rissen clone alongside seasonal shifts in secondary serotypes and resistance phenotypes. The markedly higher prevalence at downstream sampling locations, particularly in retail markets during the summer and rainy seasons, emphasizes how seasonal conditions interact with regional infrastructure limitations to amplify cross-contamination risks after slaughter. The widespread detection of complex MDR profiles, including ESBL and PMQR determinants, across multiple sample matrices further demonstrates the stability of these resistance reservoirs within the pork supply chain. Collectively, these findings underscore the need for targeted interventions that integrate season-specific hygiene control, improvements in market-level cold-chain management, and continuous genomic surveillance of dominant clones, particularly *S.* Rissen, to reduce the risk of MDR *Salmonella* entering the human food chain.

### Conclusion

This study reveals that *Salmonella* contamination and antimicrobial resistance (AMR) in the pork production chain are shaped by strong seasonal pressures and downstream amplification during distribution. Summer and the rainy season present the highest contamination risk, while winter is characterized by marked serotype convergence dominated by *S.* Rissen. These seasonal shifts have direct public health implications, as they signal predictable periods in which consumers are more likely to encounter contaminated or drug-resistant pork products. AMR patterns also varied significantly by season and serotype, with *S.* Rissen consistently exhibiting the broadest multidrug-resistant (MDR) profiles. Retail pork contained the most complex MDR combinations, indicating that resistance accumulates as products move through the supply chain—highlighting a critical exposure point for consumers. PFGE and phylogenetic analyses showed that similar clones circulate across slaughterhouses and markets, suggesting persistent transmission routes that enable MDR lineages to spread across seasons and processing stages. These findings emphasize the need for continuous genomic surveillance to identify clonal persistence, track expansion of high-risk MDR serotypes, and detect upstream control failures before contaminated products reach retail environments.

Collectively, this work provides evidence-based guidance for strengthening seasonal risk assessment, improving slaughter-to-retail hygiene practices, and integrating genomic monitoring into routine food safety surveillance to reduce public health risks.

## SUPPLEMENTAL MATERIAL

Supplemental material aem.00057-26-s0001.docx Tables S1 to S3.

ASM does not own the copyrights to Supplemental Material that may be linked to, or accessed through, an article. The authors have granted ASM a non-exclusive, world-wide license to publish the Supplemental Material files. Please contact the corresponding author directly for reuse.
