# Supplementary material for: Seasonal impacts on Salmonella prevalence and antimicrobial resistance in the pork food chain of northeastern Thailand
Source: Appl Environ Microbiol. 2026 Jun 2;92(7):e00057-26. doi: 10.1128/aem.00057-26 (PMC13390342; doi:10.1128/aem.00057-26)
Supplement: Supplemental material — Tables S1 to S3. [file aem.00057-26-s0001.docx]

**Supplemental material**

Table S1. Pairwise 2×2 chi-square analyses for seasonal comparison of *Salmonella* prevalence in carcass, feces, and pork samples.

| **Sample Category** | Season | Positive/Negative | *χ^2^* | *P*-value |
| --- | --- | --- | --- | --- |
| Carcass | Summer VS Rainy | 13/102 VS 13/87 | 0.0 | 1 |
|  | Summer VS Winter | 13/102 VS 4/96 | 2.4 | 0.12 |
|  | RainyVS Winter | 13/87 VS 4/97 | 2.7 | 0.103 |
| Feces | Summer VS Rainy | 56/55 VS 34/66 | 5.8 | 0.016 |
|  | Summer VS Winter | 56/55 VS 29/71 | 10.1 | ≤0.001 |
|  | RainyVS Winter | 34/66 VS 29/71 | 0.6 | 0.45 |
| Pork | Summer VS Rainy | 43/14 VS 73/24 | ≤0.001 | 0.99 |
|  | Summer VS Winter | 43/14 VS 64/53 | 5.0 | 0.025 |
|  | RainyVS Winter | 73/24 VS 64/53 | 6.8 | 0.009 |

Table S2: 2×2 Contingency Table of *S.*Rissen Across Different Seasons

| Season | Positive/Negative | df | *χ^2^* | *P*-value |
| --- | --- | --- | --- | --- |
| Summer VS Rainy | 25/87 VS 42/78 | 1 | 4.46 | 0.035 |
| Summer VS Winter | 25/87 VS 52/45 | 1 | 22.09 | ≤0.001 |
| RainyVS Winter | 42/78 VS 52/45 | 1 | 7.46 | 0.185 |

Table S3: Seasonal profiles of MDR patterns among *Salmonella* isolates

| **Summer** | | | **Rainy** | | | **Winter** | | |
| --- | --- | --- | --- | --- | --- | --- | --- | --- |
| Part1 | CHL/CAZ/AMP | 12.50% | Part1 | AMP/TET | 30.25% | part 1 | AMP/TET/STR/TMP/SMX | 13.40% |
| Part2 | CHL/CAZ/AMP/GEN/TET/STR | 11.61% | Part2 | CHL/AMP/GEN/TET/STR/TMP/SMX/CRO | 15.13% | part 2 | AMP/TET/STR | 13.40% |
| Part3 | AMP/TET/STR/TMP/SMX | 11.61% | Part3 | AMP/TET/STR | 14.29% | part 3 | AMP/TET/TMP/SMX | 10.31% |
| Part4 | AMP/STR | 10.71% | Part4 | CHL/CAZ/AMP/GEN/TET/STR/TMP/SMX/CRO | 13.45% | part 4 | CHL/AMP/GEN/TET/STR | 5.15% |
| Part5 | CHL/CAZ/AMP/GEN/TET/STR/TMP/SMX/CRO | 9.82% | Part5 | CHL/CAZ/AMP | 6.72% | part 5 | AMP/TET | 4.12% |
| Part6 | AMP/STR/TMP/SMX | 6.25% | Part6 | AMP/STR | 3.36% | part 6 | AMP/STR/TMP/SMX | 3.09% |
| Part7 | AMP/TET/STR | 6.25% | Part7 | CHL/AMP/TET/STR/TMP/SMX/CRO | 0.84% | part 7 | AMP/STR | 2.06% |
| Part8 | CHL/CTX/CAZ/AMP/GEN/STR/TMP/SMX/CRO | 3.57% | Part8 | CHL/AMP/GEN/TET/STR/CRO | 0.84% | part 8 | TET/STR | 2.06% |
| Part9 | CHL/AMP/STR/TMP/SMX | 3.57% | Part9 | AMP/TET/TMP/SMX | 0.84% | part 9 | CHI/CTX/CAZ/AMX/AMP/GEN/TET/STR/TMP/SMX/CRO/NA | 1.03% |
| Part10 | AMP/STR/NA | 0.89% | Part10 | TMP/SMX | 0.84% | part 10 | CHL/CTX/CAZ/AMP/TET/STR/TMP/SMX/CRO | 1.03% |
|  |  |  |  |  |  | part 11 | CHL/CTX/CAZ/AMP/TET/STR/CRO | 1.03% |
|  |  |  |  |  |  | part 12 | CHL/APM/TET/STR | 1.03% |
|  |  |  |  |  |  | part 13 | AMX/AMP/TET/STR/TMP/SMX | 1.03% |
|  |  |  |  |  |  | part 14 | AMX/AMP | 1.03% |
|  |  |  |  |  |  | part 15 | AMP/DEN | 1.03% |
|  |  |  |  |  |  | part 16 | AMP/TET/STR/NA | 1.03% |
|  |  |  |  |  |  | part 17 | AMP/TMP/SMX | 1.03% |
|  |  |  |  |  |  | part 18 | TET/STR/TMP/SMX | 1.03% |
